# (Biphenyl-4-yl)(phen­yl)methanone

**DOI:** 10.1107/S1600536810010676

**Published:** 2010-03-27

**Authors:** Aamer Saeed, Shahid Ameen Samra, Madiha Irfan, Michael Bolte

**Affiliations:** a Department of Chemistry, Quaid-i-Azam University, Islamabad 45320, Pakistan; b Kohat University of Science and Technology (KUST), Kohat, 26000, NWFP, Pakistan; c Institut für Anorganische Chemie, J. W. Goethe-Universität Frankfurt, Max-von-Laue-Strasse 7, 60438 Frankfurt/Main, Germany

## Abstract

In the title compound, C_19_H_14_O, the dihedral angle between the two aromatic rings of the biphenyl residue is 8.0 (3)° and the dihedral angle between the two rings connected by the carbonyl C atom is 51.74 (18)°. There are no short C—H⋯O contacts in the crystal structure.

## Related literature

For applications of the title compound, see: Kucybala & Wrzyszczynski (2002 ▶); van der Velden *et al.* (1980 ▶).
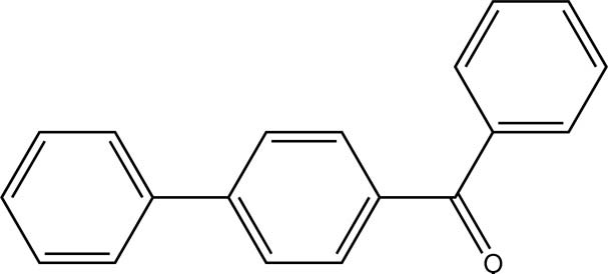


## Experimental

### 

#### Crystal data

C_19_H_14_O
*M* *_r_* = 258.30
Orthorhombic, 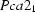

*a* = 6.1445 (4) Å
*b* = 7.4298 (7) Å
*c* = 29.014 (2) Å
*V* = 1324.56 (18) Å^3^
*Z* = 4
Mo *K*α radiation
μ = 0.08 mm^−1^
*T* = 173 K
0.32 × 0.29 × 0.12 mm

#### Data collection

Stoe IPDS II two-circle diffractometer
6073 measured reflections
1265 independent reflections
1193 reflections with *I* > 2σ(*I*)
*R*_int_ = 0.071

#### Refinement

*R*[*F* ^2^ > 2σ(*F* ^2^)] = 0.077
*wR*(*F* ^2^) = 0.182
*S* = 1.13
1265 reflections
182 parameters
1 restraint
H-atom parameters constrained
Δρ_max_ = 0.38 e Å^−3^
Δρ_min_ = −0.41 e Å^−3^

### 

Data collection: *X-AREA* (Stoe & Cie, 2001 ▶); cell refinement: *X-AREA*; data reduction: *X-AREA*; program(s) used to solve structure: *SHELXS97* (Sheldrick, 2008 ▶); program(s) used to refine structure: *SHELXL97* (Sheldrick, 2008 ▶); molecular graphics: *XP* (Sheldrick, 2008 ▶); software used to prepare material for publication: *SHELXL97*.

## Supplementary Material

Crystal structure: contains datablocks global, I. DOI: 10.1107/S1600536810010676/hb5370sup1.cif

Structure factors: contains datablocks I. DOI: 10.1107/S1600536810010676/hb5370Isup2.hkl

Additional supplementary materials:  crystallographic information; 3D view; checkCIF report

## Figures and Tables

**Fig. 1.**
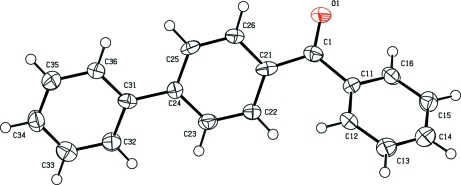
Molecular structure of title compound. Displacement ellipsoids are drawn at the 50% probability level.

## supplementary crystallographic information

### Comment

Various biphenyl derivatives are used in the synthesis of pharmaceuticals, antifungal agents like bifonazole, optical brightening agents, dyes and polychlorinated biphenyls (PCBs). PCBs are used as heat-transfer agents, as electric insulators and are environmental pollutants causing carcinogenesis. 4-Phenylbenzophenone (4-benzoylbiphenyl) is used in luminescence chemistry, spectrophotometric analysis, molecular chemistry, and as a stating material for organometallic-complexes. 4-Benzoylbiphenyl is the main photoproduct of the photodecomposition of photoinitiator *N*-[(4-benzoyl)benzene sulfonyl]benzenesulfonamide in an aqueous solution (Kucybala & Wrzyszczynski 2002). Phosphorescence and optically detected magnetic resonance (ODMR) experiments are reported for the lowest excited triplet state of 4-benzoylbiphenyl and related compounds. These compounds have been studied in single-crystal form and have a lowest m* triplet state (van der Velden *et al.*, 1980).

In the title compound, C_19_H_14_O, the dihedral angle between the two aromatic rings of the biphenyl residue is 8.0 (3)° and the dihedral angle between the two rings connected by the carbonyl C atom is 51.74 (18)°. There are no short C—H···O contacts.

### Experimental

Biphenyl (11 mmol) in chloroform (5 ml) was added slowly to a stirred mixture of anhydrous aluminum chloride (4.5 g) and benzoyl chloride (11.5 mmol) in dry chloroform (15 ml), in an ice bath and reaction mixture stirred for half hour. it was further stirred at room temperature for 3 h. Upon completion reaction mixture was diluted with water, extracted with dichloromethane and concentrated. Recrystallization from ethanol afforded (I) in 87% yield as colourless plates: Anal. calcd. for C_19_H_14_O: C, 88.34; H, 5.46%; found: C, 88.41; H, 5.61%.

### Refinement

Due to the absence of anomalous scatterers, the 1201 Friedel pairs were merged before refinement. H atoms were found in a difference map, but they were refined with fixed individual isotropic displacement parameters [*U*_iso_(H) = 1.2*U*_eq_(C)] using a riding model, with C—H = 0.95 Å.

### Crystal data

**Table:**

C_19_H_14_O	*F*(000) = 544
*M**_r_* = 258.30	*D*_x_ = 1.295 Mg m^−^^3^
Orthorhombic, *P**c**a*2_1_	Mo *K*α radiation, λ = 0.71073 Å
Hall symbol: P 2c -2ac	Cell parameters from 4666 reflections
*a* = 6.1445 (4) Å	θ = 2.8–25.9°
*b* = 7.4298 (7) Å	µ = 0.08 mm^−^^1^
*c* = 29.014 (2) Å	*T* = 173 K
*V* = 1324.56 (18) Å^3^	Plate, colourless
*Z* = 4	0.32 × 0.29 × 0.12 mm

### Data collection

**Table:**

Stoe IPDS II two-circle diffractometer	1193 reflections with *I* > 2σ(*I*)
Radiation source: fine-focus sealed tube	*R*_int_ = 0.071
graphite	θ_max_ = 25.6°, θ_min_ = 2.7°
ω scans	*h* = −6→7
6073 measured reflections	*k* = −9→8
1265 independent reflections	*l* = −35→35

### Refinement

**Table:**

Refinement on *F*^2^	Secondary atom site location: difference Fourier map
Least-squares matrix: full	Hydrogen site location: inferred from neighbouring sites
*R*[*F*^2^ > 2σ(*F*^2^)] = 0.077	H-atom parameters constrained
*wR*(*F*^2^) = 0.182	*w* = 1/[σ^2^(*F*_o_^2^) + (0.0331*P*)^2^ + 3.7315*P*] where *P* = (*F*_o_^2^ + 2*F*_c_^2^)/3
*S* = 1.13	(Δ/σ)_max_ < 0.001
1265 reflections	Δρ_max_ = 0.38 e Å^−^^3^
182 parameters	Δρ_min_ = −0.41 e Å^−^^3^
1 restraint	Extinction correction: *SHELXL97* (Sheldrick, 2008), Fc^*^=kFc[1+0.001xFc^2^λ^3^/sin(2θ)]^-1/4^
Primary atom site location: structure-invariant direct methods	Extinction coefficient: 0.032 (5)

### Special details

**Table:**

Geometry. All esds (except the esd in the dihedral angle between two l.s. planes) are estimated using the full covariance matrix. The cell esds are taken into account individually in the estimation of esds in distances, angles and torsion angles; correlations between esds in cell parameters are only used when they are defined by crystal symmetry. An approximate (isotropic) treatment of cell esds is used for estimating esds involving l.s. planes.
Refinement. Refinement of *F*^2^ against ALL reflections. The weighted *R*-factor wR and goodness of fit *S* are based on *F*^2^, conventional *R*-factors *R* are based on *F*, with *F* set to zero for negative *F*^2^. The threshold expression of *F*^2^ > σ(*F*^2^) is used only for calculating *R*-factors(gt) etc. and is not relevant to the choice of reflections for refinement. *R*-factors based on *F*^2^ are statistically about twice as large as those based on *F*, and *R*- factors based on ALL data will be even larger.

### Fractional atomic coordinates and isotropic or equivalent isotropic displacement parameters (Å^2^)

**Table:**

	*x*	*y*	*z*	*U*_iso_*/*U*_eq_	
C1	0.9334 (9)	0.2473 (12)	0.5985 (3)	0.0345 (14)	
O1	1.1328 (7)	0.2424 (10)	0.5980 (2)	0.0489 (14)	
C11	0.8158 (12)	0.2442 (9)	0.6443 (2)	0.0329 (18)	
C12	0.6194 (12)	0.3247 (12)	0.6502 (3)	0.045 (2)	
H12	0.5463	0.3761	0.6245	0.055*	
C13	0.5235 (12)	0.3323 (11)	0.6943 (2)	0.0418 (19)	
H13	0.3865	0.3890	0.6988	0.050*	
C14	0.6325 (14)	0.2562 (12)	0.7305 (3)	0.050 (2)	
H14	0.5711	0.2643	0.7605	0.060*	
C15	0.8288 (12)	0.1680 (9)	0.7250 (2)	0.0351 (17)	
H15	0.8991	0.1124	0.7505	0.042*	
C16	0.9205 (12)	0.1628 (11)	0.6813 (2)	0.0363 (18)	
H16	1.0557	0.1033	0.6767	0.044*	
C21	0.8136 (11)	0.2548 (7)	0.5540 (2)	0.0265 (15)	
C22	0.6040 (10)	0.1827 (8)	0.5471 (2)	0.0243 (14)	
H22	0.5254	0.1355	0.5726	0.029*	
C23	0.5128 (11)	0.1800 (10)	0.5041 (2)	0.0316 (16)	
H23	0.3734	0.1266	0.5003	0.038*	
C24	0.6188 (11)	0.2540 (7)	0.4649 (2)	0.0222 (14)	
C25	0.8263 (11)	0.3235 (10)	0.4727 (2)	0.0344 (17)	
H25	0.9029	0.3739	0.4474	0.041*	
C26	0.9260 (11)	0.3227 (11)	0.5153 (2)	0.0341 (17)	
H26	1.0698	0.3678	0.5186	0.041*	
C31	0.5192 (10)	0.2543 (8)	0.4186 (2)	0.0285 (14)	
C32	0.3245 (11)	0.1634 (9)	0.4098 (2)	0.0330 (16)	
H32	0.2518	0.1041	0.4344	0.040*	
C33	0.2347 (12)	0.1577 (9)	0.3659 (3)	0.0370 (16)	
H33	0.1035	0.0931	0.3608	0.044*	
C34	0.3348 (12)	0.2456 (10)	0.3295 (2)	0.0380 (17)	
H34	0.2726	0.2437	0.2995	0.046*	
C35	0.5263 (12)	0.3357 (11)	0.3378 (2)	0.0415 (19)	
H35	0.5977	0.3954	0.3131	0.050*	
C36	0.6174 (11)	0.3412 (10)	0.3814 (2)	0.0360 (17)	
H36	0.7492	0.4054	0.3861	0.043*	

### Atomic displacement parameters (Å^2^)

**Table:**

	*U*^11^	*U*^22^	*U*^33^	*U*^12^	*U*^13^	*U*^23^
C1	0.022 (3)	0.044 (4)	0.037 (3)	0.001 (3)	−0.001 (4)	−0.002 (3)
O1	0.023 (2)	0.086 (4)	0.037 (2)	−0.007 (3)	0.004 (3)	−0.007 (2)
C11	0.026 (4)	0.047 (5)	0.026 (4)	−0.006 (3)	0.004 (3)	−0.001 (3)
C12	0.026 (4)	0.077 (6)	0.033 (4)	−0.006 (4)	−0.006 (3)	0.003 (4)
C13	0.032 (4)	0.055 (5)	0.038 (4)	0.005 (4)	0.003 (3)	−0.006 (4)
C14	0.039 (5)	0.074 (7)	0.038 (5)	−0.003 (4)	0.005 (4)	−0.006 (4)
C15	0.041 (4)	0.031 (4)	0.033 (4)	−0.006 (3)	−0.002 (3)	−0.003 (3)
C16	0.025 (4)	0.049 (5)	0.034 (4)	0.000 (3)	−0.005 (3)	−0.009 (4)
C21	0.023 (3)	0.014 (3)	0.042 (4)	−0.003 (2)	0.010 (3)	−0.006 (3)
C22	0.025 (3)	0.017 (3)	0.031 (4)	−0.003 (3)	0.001 (3)	−0.002 (3)
C23	0.023 (3)	0.038 (4)	0.034 (4)	0.000 (3)	0.004 (3)	−0.004 (3)
C24	0.025 (3)	0.018 (3)	0.024 (3)	0.005 (2)	0.000 (3)	−0.003 (3)
C25	0.025 (4)	0.046 (4)	0.032 (4)	−0.008 (3)	0.011 (3)	0.001 (3)
C26	0.020 (3)	0.047 (5)	0.035 (4)	−0.002 (3)	0.001 (3)	0.004 (3)
C31	0.022 (3)	0.028 (3)	0.035 (4)	−0.003 (3)	0.001 (3)	−0.003 (3)
C32	0.036 (4)	0.028 (3)	0.034 (4)	−0.009 (3)	−0.001 (3)	0.002 (3)
C33	0.035 (3)	0.032 (4)	0.044 (4)	0.001 (3)	−0.003 (3)	−0.006 (3)
C34	0.038 (4)	0.048 (5)	0.028 (3)	0.010 (3)	−0.006 (3)	−0.002 (3)
C35	0.034 (4)	0.057 (5)	0.033 (4)	0.003 (4)	0.004 (3)	−0.001 (3)
C36	0.025 (3)	0.053 (4)	0.030 (3)	−0.006 (3)	0.000 (3)	−0.003 (3)

### Geometric parameters (Å, °)

**Table:**

C1—O1	1.226 (7)	C23—C24	1.421 (9)
C1—C21	1.489 (10)	C23—H23	0.9500
C1—C11	1.512 (10)	C24—C25	1.393 (9)
C11—C12	1.357 (11)	C24—C31	1.476 (9)
C11—C16	1.391 (11)	C25—C26	1.380 (10)
C12—C13	1.412 (10)	C25—H25	0.9500
C12—H12	0.9500	C26—H26	0.9500
C13—C14	1.368 (11)	C31—C32	1.398 (9)
C13—H13	0.9500	C31—C36	1.395 (9)
C14—C15	1.382 (11)	C32—C33	1.388 (10)
C14—H14	0.9500	C32—H32	0.9500
C15—C16	1.388 (10)	C33—C34	1.387 (10)
C15—H15	0.9500	C33—H33	0.9500
C16—H16	0.9500	C34—C35	1.375 (10)
C21—C22	1.409 (9)	C34—H34	0.9500
C21—C26	1.410 (10)	C35—C36	1.385 (9)
C22—C23	1.368 (9)	C35—H35	0.9500
C22—H22	0.9500	C36—H36	0.9500
			
O1—C1—C21	119.0 (8)	C24—C23—H23	118.8
O1—C1—C11	119.3 (8)	C25—C24—C23	115.7 (6)
C21—C1—C11	121.8 (5)	C25—C24—C31	121.8 (6)
C12—C11—C16	120.4 (7)	C23—C24—C31	122.5 (6)
C12—C11—C1	121.9 (7)	C26—C25—C24	123.4 (6)
C16—C11—C1	117.6 (7)	C26—C25—H25	118.3
C11—C12—C13	120.2 (7)	C24—C25—H25	118.3
C11—C12—H12	119.9	C25—C26—C21	119.8 (6)
C13—C12—H12	119.9	C25—C26—H26	120.1
C14—C13—C12	118.4 (7)	C21—C26—H26	120.1
C14—C13—H13	120.8	C32—C31—C36	116.8 (6)
C12—C13—H13	120.8	C32—C31—C24	121.5 (6)
C13—C14—C15	122.3 (8)	C36—C31—C24	121.7 (5)
C13—C14—H14	118.8	C33—C32—C31	121.6 (6)
C15—C14—H14	118.8	C33—C32—H32	119.2
C14—C15—C16	118.2 (8)	C31—C32—H32	119.2
C14—C15—H15	120.9	C34—C33—C32	120.6 (7)
C16—C15—H15	120.9	C34—C33—H33	119.7
C15—C16—C11	120.4 (7)	C32—C33—H33	119.7
C15—C16—H16	119.8	C35—C34—C33	118.4 (7)
C11—C16—H16	119.8	C35—C34—H34	120.8
C22—C21—C26	118.1 (7)	C33—C34—H34	120.8
C22—C21—C1	124.1 (6)	C34—C35—C36	121.4 (7)
C26—C21—C1	117.5 (6)	C34—C35—H35	119.3
C23—C22—C21	120.6 (6)	C36—C35—H35	119.3
C23—C22—H22	119.7	C35—C36—C31	121.3 (6)
C21—C22—H22	119.7	C35—C36—H36	119.4
C22—C23—C24	122.4 (6)	C31—C36—H36	119.4
C22—C23—H23	118.8		
			
O1—C1—C11—C12	150.5 (9)	C22—C23—C24—C25	2.5 (9)
C21—C1—C11—C12	−29.9 (12)	C22—C23—C24—C31	−178.9 (6)
O1—C1—C11—C16	−26.5 (13)	C23—C24—C25—C26	−0.2 (10)
C21—C1—C11—C16	153.1 (7)	C31—C24—C25—C26	−178.9 (7)
C16—C11—C12—C13	2.3 (12)	C24—C25—C26—C21	−2.3 (11)
C1—C11—C12—C13	−174.5 (8)	C22—C21—C26—C25	2.5 (10)
C11—C12—C13—C14	−0.4 (12)	C1—C21—C26—C25	176.5 (7)
C12—C13—C14—C15	−2.0 (13)	C25—C24—C31—C32	171.3 (6)
C13—C14—C15—C16	2.3 (12)	C23—C24—C31—C32	−7.3 (9)
C14—C15—C16—C11	−0.2 (11)	C25—C24—C31—C36	−7.0 (9)
C12—C11—C16—C15	−2.1 (11)	C23—C24—C31—C36	174.4 (6)
C1—C11—C16—C15	174.9 (7)	C36—C31—C32—C33	0.9 (9)
O1—C1—C21—C22	150.3 (8)	C24—C31—C32—C33	−177.5 (6)
C11—C1—C21—C22	−29.3 (11)	C31—C32—C33—C34	−1.1 (11)
O1—C1—C21—C26	−23.3 (12)	C32—C33—C34—C35	1.0 (11)
C11—C1—C21—C26	157.1 (7)	C33—C34—C35—C36	−0.7 (11)
C26—C21—C22—C23	−0.3 (9)	C34—C35—C36—C31	0.5 (12)
C1—C21—C22—C23	−173.9 (7)	C32—C31—C36—C35	−0.6 (10)
C21—C22—C23—C24	−2.2 (10)	C24—C31—C36—C35	177.8 (6)

## References

[bb1] Kucybala, K. & Wrzyszczynski, A. (2002). *J. Photochem. Photobiol. A*, **153**, 109–112.

[bb2] Sheldrick, G. M. (2008). *Acta Cryst.* A**64**, 112–122. 10.1107/S0108767307043930 18156677

[bb3] Stoe & Cie (2001). *X-AREA* Stoe & Cie, Darmstadt, Germany.

[bb4] Velden, G. P. M. de van der, Boer, E. & Veeman, W. S. (1980). *J. Phys. Chem.* **84**, 2634–2641.

